# Caries of Left Temporal Bone

**Published:** 1847-06

**Authors:** 


*Caries of Left Temporal Bone.*—No ward exhibited to the Pathological Society of London, a specimen of the disease in which the condition of the bone had implicated this temporo-maxillary articulation, which was destroyed, with the exception of the fibro-cartilage. Opposite the carious anterior wall of the petrous portion, an ulcer, with smooth borders, about the size of a threepenny-piece, was observed in the dura mater. The entire surface of the left hemisphere of the brain was covered over by a layer of greenish-brown lymph; and a large quantity of arterial blood, apparently from a branch of the middle meningeal, which had ulcerated, was effused on its anterior, lateral, and upper portions, and on the corresponding parts of the dura mater. The condition of the brain and dura mater was illustrated by a drawing. The preparation was taken from a girl aged sixteen, who had been treated in the London Hospital, by Drs. Frampton and Fraser, for acute endocarditis.

On her admission, the purulent discharge from the ear was not sufficiently great to attract much attention, and she never complained of any head symptoms. As soon, however as she was placed under the influence of mercury, the discharge greatly augmented; and sixteen days after admission, the soft parts of the external ear began to slough. The discharge increased daily in quantity, and ultimately became very profuse and horribly fœtid. About six weeks after her admission, profuse hæmorrhage came on at two distinct periods, and she died comatose, no paralysis or loss of consciousness having been previously remarked.

